# The Key Metabolic Network and Genes Regulating the Fresh Fruit Texture of Jujube (*Ziziphus jujuba* Mill.) Revealed via Metabolomic and Transcriptomic Analysis

**DOI:** 10.3390/plants12112087

**Published:** 2023-05-24

**Authors:** Shuang Song, Juan Jin, Meiyu Li, Decang Kong, Ming Cao, Xue Wang, Yingyue Li, Xuexun Chen, Xiuli Zhang, Xiaoming Pang, Wenhao Bo, Qing Hao

**Affiliations:** 1 The State Key Laboratory of Genetic Improvement and Germplasm Innovation of Crop Resistance in Arid Desert Regions (Preparation), Key Laboratory of Genome Research and Genetic Improvement of Xinjiang Characteristic Fruits and Vegetables, Institute of Horticulture Crops, Xinjiang Academy of Agricultural Sciences, Urumqi 830091, China; shuangsong17@163.com (S.S.); jinjuan316@126.com (J.J.); 2 Henan Key Laboratory of Germplasm Innovation and Utilization of Eco-Economic Woody Plant, Pingdingshan University, Pingdingshan 467000, China; meiyulily@163.com; 3 National Foundation for Improved Cultivar of Chinese Jujube, Cangzhou 061000, China; kongdecang@126.com (D.K.); i2008caoming@126.com (M.C.); 4 State Key Laboratory of Tree Genetics and Breeding, National Engineering Research Center of Tree Breeding and Ecological Restoration, Key Laboratory of Genetics and Breeding in Forest Trees and Ornamental Plants, Ministry of Education, College of Biological Sciences and Biotechnology, Beijing Forestry University, Beijing 100083, China; wangxue1660@163.com (X.W.); yingyueli@bjfu.edu.cn (Y.L.); xmpang@163.com (X.P.); 5 Bureau of Forestry of Aohan, Chifeng 028000, China; xuexunchen@163.com (X.C.); nmcxx2008@163.com (X.Z.)

**Keywords:** fruit texture, metabolomic, transcriptomic, combined analysis, galactose metabolism, jujube (*Ziziphus jujuba*)

## Abstract

The texture of fresh jujube fruit is related to its popularity and commercial value. The metabolic networks and essential genes that regulate the texture of jujube (*Ziziphus jujuba*) fruit are still unknown. In this study, two jujube cultivars with significantly different textures were selected by a texture analyzer. The four developmental stages of the exocarp and mesocarp of jujube fruit were studied separately using metabolomic and transcriptomic analyses. Differentially accumulated metabolites were enriched in several critical pathways related to cell wall substance synthesis and metabolism. Transcriptome analysis confirmed this by finding enriched differential expression genes in these pathways. Combined analysis showed that ‘Galactose metabolism’ was the most overlapping pathway in two omics. Genes such as *β-Gal*, *MYB* and *DOF* may affect fruit texture by regulating cell wall substances. Overall, this study provides an essential reference for the establishment of texture-related metabolic and gene networks of jujube fruit.

## 1. Introduction

Chinese jujube (*Ziziphus jujuba* Mill.), one of the native critical non-wood forest tree species of China, has been cultivated for over 3000 years [1]. Jujube fruit is rich in vitamin C and vitamin P and can be used to make candied jujube, dried jujube, jujube wine and preserved fruit. Jujube fruit has a crisp taste, refreshing juice, and a sweet and sour taste, making it a highly popular fruit among consumers in China [2].

The quality of fresh jujube fruit is the main factor affecting its competitiveness in the market. Fruit texture, together with the sugar to acid ratio and fruit size, constitute an important index for evaluating the quality of fresh jujube fruit; thus, fruit texture is one of the main drivers of consumer purchasing decisions [3]. It affects consumer acceptance, transportability, disease resistance, and shelf life. For fresh jujube fruits, both the exocarp and mesocarp are consumed together, and thus the texture of the exocarp is as crucial as that of the mesocarp. A texture analyzer is usually chosen to evaluate fruit texture [4]. There are some studies on the texture detection of fresh jujube fruit using the texture analyzer [5,6,7]. The studies on jujube fruit texture have mainly focused on the flesh [7]; therefore, studying the texture of jujube fruit concerning both the exocarp and mesocarp is of great practical significance.

Fruit texture is closely related to cell walls, especially the mechanical strength of cell walls and intercellular turgor pressure [8]. The primary wall of plants is composed of solid cellulose microfilaments embedded in a network of hemicellulose and pectin crosslinks, as well as a small number of structural proteins [9]. Pectin polysaccharides strengthen the gel skeleton that interacts with cellulose by forming what is known as an ‘egg box structure’, a cross-linked chain of divalent calcium ions and esterified polygalacturonic acid [10]. The dissolution of pectin caused the dissolution of the intercellular layer rich in protopectin, which affected the texture of fruits [11]. Atomic force microscopy (AFM) observation showed that the fruit with thick cellulose microfilaments showed a hard, crisp, and juicy taste [12]. The differences in the structure and composition of the cell wall are the result of the cooperative action of multiple enzymes. The role of some cell wall genes in fruit texture has been well documented, such as that of *PL* and *β-Gal* [13,14]. However, much remains to be understood about the molecular regulation of fruit texture in Chinese jujube.

With the rapid development of modern biotechnology and bioinformatics, multi-omics research has become an essential part of the field of systems biology. Metabolomic and transcriptomic analysis can establish metabolite–gene correlation networks and screen candidate genes involved in metabolic pathways [15]. The combination of functional annotation and metabolic pathway analysis allows the identification of key genes and crucial pathways, which can then be used to form a network and analyze the underlying biological processes [16]. A lot of progress has been made in metabolomic and transcriptomic analysis on jujube. For example, this includes progress in the molecular mechanism of exocarp color formation of ‘Tailihong’ jujube and the changes in active components and related differential genes during the growth and development of ‘Jinsixiaozao’ [17,18]. Nevertheless, the metabolic networks and genes related to texture have never been reported

The texture of fresh jujube fruit changes from hard to crisp with moderate hardness during ripening [19]. *Z. jujuba* ‘Dongzao’ is known for its crisp texture, as well as its distinct flavor and abundant nutrients [20], while ‘P15’ has been recorded as a hard exocarp and mesocarp. The substantial difference in texture between these two cultivars prompted this study to investigate the underlying mechanism of texture. In this study, the fruit texture of these two cultivars with contrasting textures were evaluated based on a texture analyzer. Subsequently, the differences in metabolites and genes were analyzed based on metabolomic and transcriptomic data obtained from four fruit development stages. The model and correlation analysis explored the relationship between the differential metabolites and genes. This study explained the metabolic basis and molecular mechanism of texture regulation of fresh jujube fruit and identified critical metabolites that could be used to predict and quantify jujube fruit texture, which provides a reference for further genetic improvement of jujube fruit quality.

## 2. Results

### 2.1. Texture Difference between ‘Dongzao’ and ‘P15’

**Figure 1:**
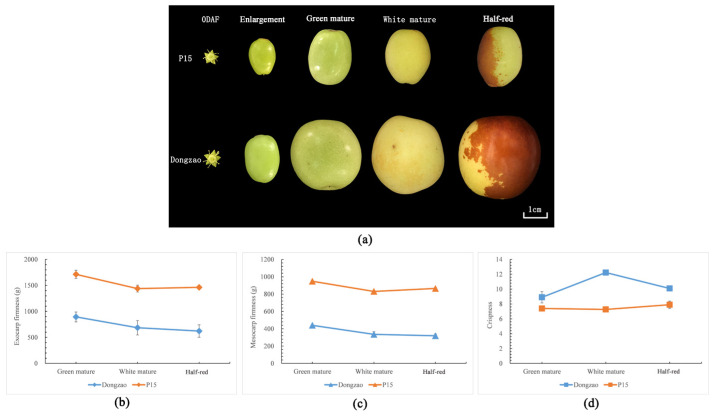
(**a**) Sampling stage of two cultivars. (**b**) The exocarp firmness of two cultivars at different stages. (**c**) The mesocarp firmness of two cultivars at different stages. (**d**) The crispness of two cultivars at different stages.

The overview of sampling stages of the two cultivars is illustrated in Figure 1a. Since the fruits were too small at the enlargement stage, fruit texture was only detected in the remaining three stages. The firmness of the exocarp and mesocarp of ‘P15’ was obviously higher than that of ‘Dongzao’ at all stages (almost three times higher) (Figure 1b). However, the crispness of ‘P15’ was lower than that of ‘Dongzao’ (Figure 1b).

### 2.2. Metabolomic Profiles

**Figure 2:**
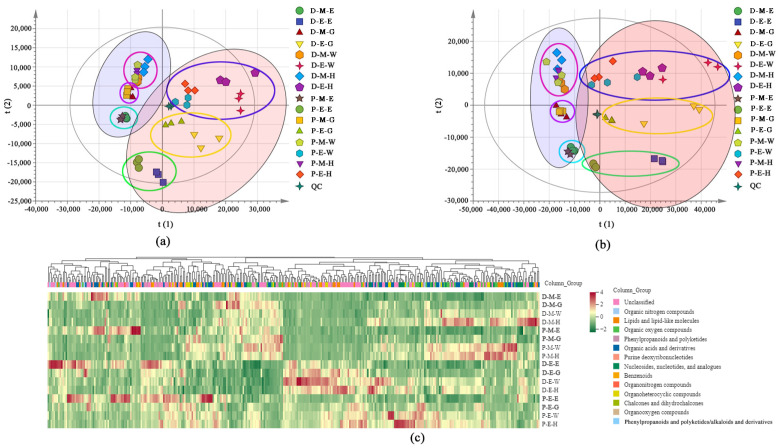
(**a**) PCA of positive-ion metabolites of all samples. (**b**) PCA of negative-ion metabolites of all samples. (**c**) Heatmap of all metabolites. D: ‘Dongzao’. P: ‘P15’. E: exocarp. M: mesocarp. E: enlargement. G: green mature stage. W: white mature stage. H: halfred stage. QC: quality control.

The overlapping comparison results of UHPLC-Q-TOF MS total ion chromatogram patterns of QC (quality control) samples are shown in Figure S1. The response intensity and retention time of each chromatographic peak are basically overlapped, which shows that the variation caused by the instrument error is small during the whole experimental process. A total of 328 metabolites were identified via non-targeted metabolomics, including 151 positive and 177 negative ions (Table S1). These metabolites were divided into 14 categories, including 56 organic acids and derivatives, 43 organic oxygen compounds, 33 lipids, lipid-like molecules, and so on (Figure S1). PCA analysis was performed on metabolites to reflect the variability between and within sample groups (Figure 2a,b). It can be seen from the figure that the QC samples are closely gathered in positive- and negative-ion modes, suggesting that the experiment of this project has good repeatability. The samples within each group were consistent, and the differences between groups were significant. Mesocarp samples were separated from exocarp samples (Figure 2a,b). Among the four developmental stages, the white mature stage and half-red stage were closely clustered, but the enlargement stage and green mature stage were separated. The expression heat map also showed various metabolites in different tissues and stages (Figure 2c).

### 2.3. DAMs Profiling in Exocarp and Mesocarp of Two Varieties at Different Development Stages

The metabolomic analysis of exocarp was divided into four comparison groups: D_E_E vs. P_E_E, D_E_G vs. P_E_G, D_E_W vs. P_E_W and D_E_H vs. P_E_H. The Venn diagram shows that 33 DAMs were screened at all stages (Figure S2a). These included D-fructose, D-galactarate, D-tagatose, and so on. The comparison results showed that the green mature stage had the most DAMs, with 22 upregulated and 49 downregulated. The half-red stage had 65 DAMs, including 18 upregulated and 47 downregulated. The enlargement stage and the white mature stage had the same number of DAMs; there were 23 upregulated and 37 downregulated at the enlargement stage, and 19 upregulated and 42 downregulated in the latter stage (Figure S2b). Information about all screened DAMs can be found in Table S2A. 

KEGG metabolic pathways were analyzed using Fisher’s precise test for DAMs in each comparison group (Figure S3). The number of metabolic pathways enriched at the enlargement stage was the least, while that of the white mature stage was the most. Among them, the pathways with significant differences in the four stages were mainly concentrated in ‘Galactose metabolism’, ‘Ascorbate and aldarate metabolism’, ‘Fatty acid biosynthesis beta-Alanine metabolism’, and ‘Fructose and mannose metabolism’ pathways.

The metabolomic analysis of the mesocarp was also divided into four comparison groups: D_M_E vs. P_M_E, D_M_G vs. P_M_G, D_M_W vs. P_M_W and D_M_H vs. P_M_H. Five metabolites were defined as DAMs at all stages, which is less than those of the exocarp (Figure S4a). The number of DAMs at each stage is shown in Figure S4b, and the details are presented in Table S2B. Some metabolites were identified as DAMs in both the exocarp and mesocarp, particularly those associated with cell wall composition, such as D-fructose, D-galactarate, D-galacturonic acid, galactinol, D-mannose, and so on. KEGG analysis showed that most DAMs were enriched in ‘Galactose metabolism’, ‘Ascorbate and aldarate metabolism’, ‘Fatty acid biosynthesis beta-Alanine metabolism’, and ‘Starch and sucrose metabolism’ pathways (Figure S5). Interestingly, the ‘Galactose metabolism’ pathway was enriched at all developmental stages in both the exocarp and mesocarp.

### 2.4. Transcriptomic Analysis in Exocarp and Mesocarp of Two Varieties at Different Development Stages

The overall sequencing quality results are shown in Table S3. Briefly, 343.12 Gb clean data were obtained. After removing low-quality reads, an average of 47,656,036 clean reads per sample were generated. The Q30 of each sample was above 93%, and the GC content was between 43.36% and 44.38%. When mapping clean reads with the reference genome ‘Junzao’, the average comparison efficiency was 87.45%. 

According to the screening criteria of DEGs, the most DEGs in the exocarp were observed at the white mature stage, with 539 genes upregulated and 1095 genes downregulated (Figure S6a). The number of DEGs at the enlargement stage was the least (448 upregulated and 732 downregulated). A comprehensive analysis of comparative groups in different stages revealed 249 DEGs found at all stages (Figure S6b). These DEGs included cellulose synthase, arabinosyl transferase, and the GATA transcription factor. 

For GO analyses, DEGs were primarily enriched in ‘Molecular Function’, such as ‘cellulose synthase activity’, ‘glycosyltransferase activity’, and ‘polysaccharide binding’ (Figure S7). ‘Biological Process’ also enriched many DEGs, for example, ‘cell wall macromolecule metabolic process’ and ‘cell wall organization or biogenesis’. According to the KEGG analysis, these DEGs were mainly enriched in ‘Galactose metabolism’, ‘Fructose and mannose metabolism’, and ‘Flavonoid biosynthesis’ pathways (Figure S8). 

The largest number of DEGs of the mesocarp were found at the white mature stage, while the smallest was observed at the green mature stage (Figure S9a). After a comprehensive analysis of all comparison groups, 241 DEGs were found in all stages (Figure S9b), including L-ascorbate oxidase, calcium-binding protein, WRKY transcription factor, etc. Of these shared DEGs, 157 were screened in both the mesocarp and exocarp. Similarly to the results of exocarp enrichment, DEGs of the mesocarp were enriched in large quantities in ‘Molecular Function’, which contained terms such as ‘Polysaccharide binding’ and ‘Cellulose synthase (UDP-forming) activity’ (Figure S10). We mapped DEGs to the KEGG database and found that these DEGs were most enriched in ‘Galactose metabolism’, ‘ABC transporters’, ‘Plant-pathogen interaction’ pathways, and so on (Figure S11).

### 2.5. ‘Galactose Metabolism’ Is the Most Closely Related Pathway between the Two-Omics Data

We performed a K-means clustering analysis to understand the expression patterns of DAMs and DEGs from the exocarp (Figure S12a,b). These DAMs and DEGs were divided into eight clusters, each displaying similar expression patterns. The expression patterns of DAMs in Class 2 and 5 were higher in the cultivar ‘Dongzao, while Class 4 and Class 6 showed higher expression in the cultivar ‘P15’. The DEGs of Class 3 consisted of 183 upregulated genes in ‘Dongzao’, and Class 2 had 136 genes that were upregulated in ‘P15’.

KEGG enrichment analyses of DAMs and DEGs at four developmental stages were compared, and the coincidence pathways at the same developmental stage were screened out (Table 1). The ‘Galactose metabolism’ pathway (map00052) coincided at all stages except for the enlargement stage. The ‘Cutin, suberine and wax biosynthesis’ pathway (map00073) and ‘ABC transporters’ pathway (map02010) coincided more than once.

The O2PLS model is often used in the integration analysis of two data groups. The load diagram in Figure S13 shows the first 25 variables that significantly influence another group, with red Xs as DAMs and blue Ys as DEGs. Details are shown in Table S4. Among these highly correlated DAM variables were D-galactarate, galactinol, dehydroascorbic acid, D-tagatose, alpha-D-glucose, and sucrose, which are enriched in ‘Ascorbate and aldarate metabolism’, ‘Galactose metabolism’, ‘Fructose and mannose metabolism’, ‘Pentose and glucuronate interconversions’ and ‘Starch and sucrose metabolism’ pathways. These pathways include the degradation of cell wall components such as pectin and cellulose and some synthetic precursors of cell wall polysaccharides or necessary factors affecting their degradation. However, DEG variables include many unannotated genes, and other annotated genes are not related to fruit texture. Only one MYB transcription factor (evm.model.Contig11.191) may participate in fruit texture regulation.

In order to find a strong connection between gene expression patterns and metabolite accumulation, we conducted Pearson’s correlation analysis on DAMs that were enriched in the pathways previously mentioned, as well as all the DEGs. DEGs with r ≥ 0.8 and *p* ≤ 0.05 were screened out, and the expression patterns of genes related to cell wall anabolism and transcription factors were analyzed.

**Figure 3:**
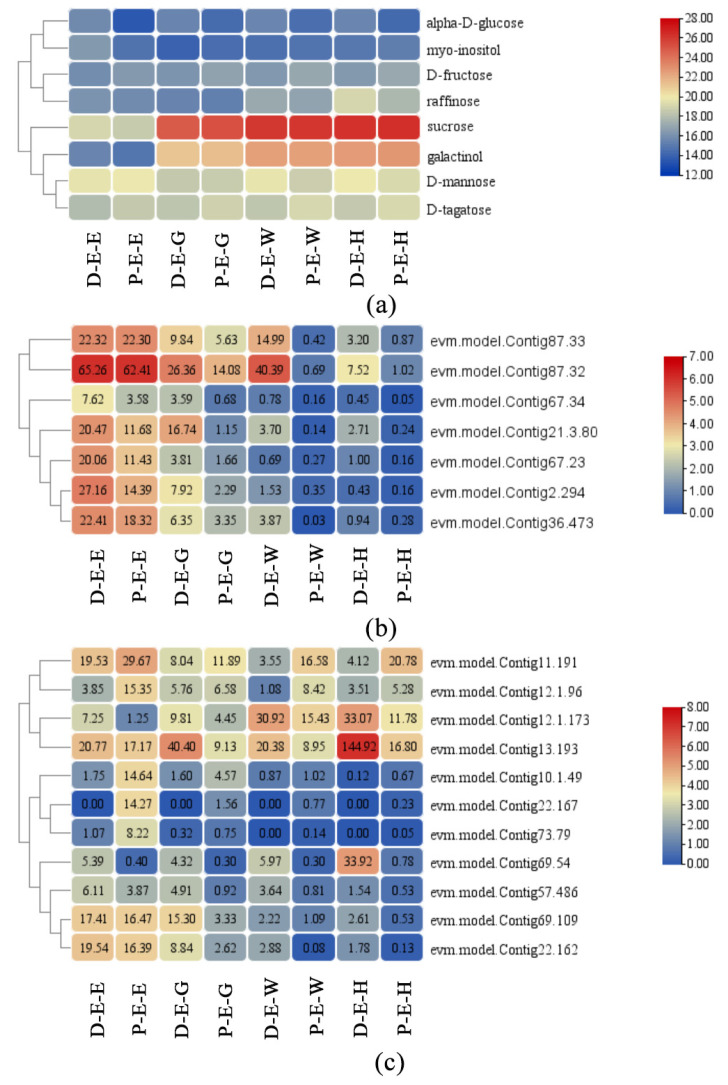
Expression levels of critical DAMs and DEGs in exocarp. (**a**) DAMs. (**b**) DEGs. (**c**) Transcription factors. D: ‘Dongzao’. P: ‘P15’. E: exocarp. M: mesocarp. E: enlargement. G: green mature stage. W: white mature stage. H: half-red stage.

The ‘Galactose metabolism’ pathway was the most closely related pathway connecting the two omics. A total of eight differential metabolites were enriched in this pathway. D-mannose, alpha-D-glucose, D-tagatose, and D-fructose were screened out in all stages. Sucrose was selected at three stages except for the half-red stage. Myo-inositol was screened out at three stages except for the enlargement stage. Galactinol was only detected at the green mature stage and raffinose was found at the half-red stage. Among them, alpha-D-glucose and raffinose were in the upregulation mode in ‘Dongzao’, while D-tagatose and D-fructose showed downregulation (Figure 3a). Some DAMs are also involved in other metabolic pathways related to cell wall polysaccharides (Table 2). For example, D-fructose has also been contained in the ‘Fructose and mannose metabolism’ pathway. L-fucose and L-rhamnose are pectin components in this pathway. The ‘Starch and sucrose metabolism’ pathway, which includes the synthesis and degradation of cellulose, also enriches D-fructose and sucrose. 

In these highly correlated DEGs, the expression patterns of several genes related to the cell wall were consistent with the texture phenotype (Table 2, Figure 3b). *β-Gal*, *PL*, and *β-Glu* are essential genes that degrade pectin, cellulose, and other polysaccharides in cell walls. These genes showed higher expression in ‘Dongzao’. There are several transcription factors in the highly correlated DEGs, such as *AP2/ERF*, *TCP*, and *DOF*. Additionally, several common transcription factors, such as *WRKY*, *bHLH*, and *GATA*, showed high correlation with many DAMs (Figure 3c). The MYB transcription factor (evm.model.Contig11.191), which was expressed higher in ‘Dongzao’, was also one of the first 25 variables in O2PLS analysis.

Subsequently, the same analyses were performed in the mesocarp. All DAMs and DEGs were divided into eight classes (Figure S14a,b). Class 1, 6, and 8 of the DAMs presented a trend of low expression in ‘Dongzao’, but Class 5 was the opposite. A total of 179 DEGs in Class 1 were expressed higher in ‘P15’ and 178 DEGs in Class 5 were expressed higher in ‘Dongzao’. Among the KEGG pathways that DAMs and DEGs enriched, ‘Galactose metabolism’ (map00052) and ‘ABC transporters’ (map02010) pathwayscoincided in the white mature stage and half-red stage. In addition, the ‘Ascorbate and aldarate metabolism’ (map00053) pathway coincided in the half-red stage (Table 3). The variables with high correlation in O2PLS analysis included UDP-D-glucose, L-threonate, D-tagatose, and D-galactarate (Figure S15, Table S5). These metabolites were enriched in ‘Ascorbate and aldarate metabolism’, ‘Galactose metabolism’, ‘Pentose and glucuronate interconversions’, and ‘Starch and sucrose metabolism’ pathways. DEG variables included one AP2/B3-like transcriptional factor (evm.model.Contig51.23), which may be related to fruit texture.

**Figure 4:**
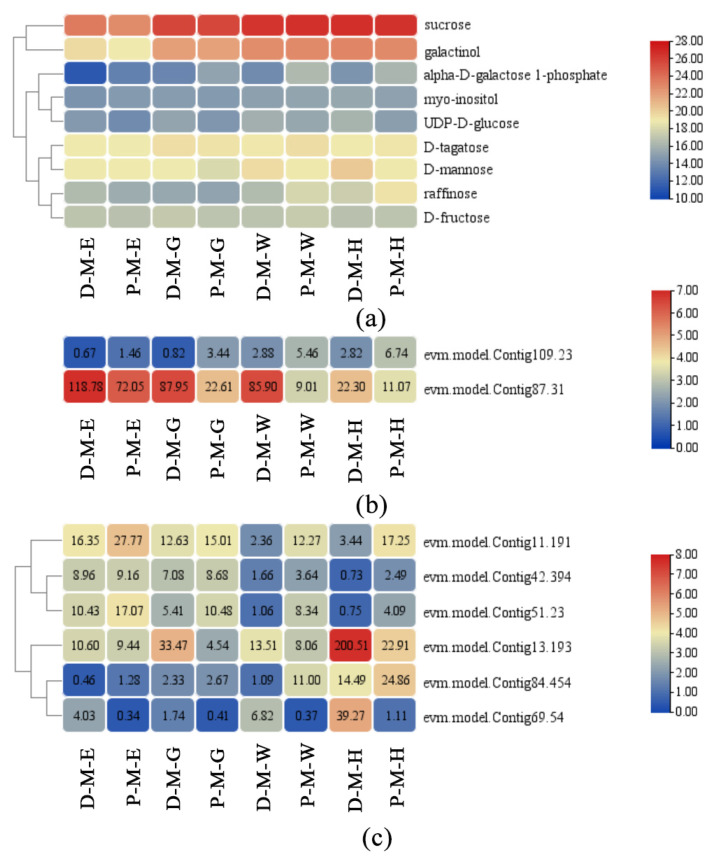
Expression levels of critical DAMs and DEGs in the mesocarp. (**a**) DAMs. (**b**) DEGs. (**c**) Transcription factors. D: ‘Dongzao’. P: ‘P15’. E: exocarp. M: mesocarp. E: enlargement. G: green mature stage. W: white mature stage. H: half-red stage.

Similarly to the findings in the exocarp, the DAMs of the mesocarp that enriched in the ‘Galactose metabolism’ pathway deserved further exploration. Raffinose, D-fructose, sucrose, galactinol, alpha-D-galactose 1-phosphate, D-tagatose, myo-inositol, D-mannose, and uridine diphosphate glucose (UDP-D-glucose) were included in this pathway. D-mannose and UDP-D-glucose were accumulated higher in ‘Dongzao’, and alpha-D-galactose 1-phosphate was accumulated higher in ‘P15’ (Figure 4a). 

In the correlation analysis, we focus on the DEGs with r ≥ 0.8 and *p* ≤ 0.05. The expression trend of the cellulose synthesis and metabolism genes (evm.model.Contig109.23 and evm.model.Contig87.31), which were highly related to α-D-galactose 1-phosphate, were consistent with the mesocarp texture in the two cultivars (Table 4, Figure 4b). Some transcription factors were also screened (Table 4, Figure 4c). These transcription factors correlate with DAMs in the ‘Galactose metabolism’ pathway. Among them, three genes (evm.model.Contig11.191, evm.model.Contig69.54, and evm.model.Contig13.193) were also identified in the screening of DEGs in the exocarp, and they had the same expression pattern in two tissues (Table 2 and Table 4, Figure 3c and Figure 4c).

## 3. Discussion

The texture of fresh jujube is an intrinsic quality of the product and a crucial factor in determining its quality [21]. However, there is still a lack of comprehensive understanding of the metabolites and gene regulatory networks affecting the texture of fresh jujube fruit. In this study, the key metabolites and genes related to different fruit textures were excavated using the combined analysis of two omics. We constructed related metabolic networks and regulations, which indicated the role of galactose metabolism related compounds in quantifying fruit texture as a quantitative index. This information serves as a useful reference for preserving jujube fruit and for breeding new varieties.

The results of the texture analyzer can clearly show the significant difference in texture between the exocarp and mesocarp of these two cultivars. Fruit texture depends on the anatomy of the plant tissue, the strength of the cell wall, and the cohesion and turgor of the middle lamella. The primary cell wall is composed of polysaccharides, a small portion of glycoproteins, and some non-carbohydrate substances. Its structure consists of solid cellulose microfibrils embedded in a cross-linked network of hemicellulose and pectin [22]. The main components of pectin are rhamnose, arabinose, and galacturonic acid. Cellulose is a chain-like β-(1, 4)-glucan. Microfibers are crystallized by intermolecular hydrogen bonds and a van der Waals force, forming a stretchable hard structure, which is closely connected to that of hemicellulose. Hemicellulose is a kind of polysaccharide with an isomeric structure, which mainly includes D-glucose, xylose, or mannose [23]. The components of these polysaccharides can be used as metabolites of great concern. Pathways related to the metabolism of these cell wall polysaccharides are of particular interest, such as ‘Pentose and glucuronate interconversions’ and ‘Starch and sucrose metabolism’ pathways.

The accumulation of metabolites was closely related to different tissues and developmental stages. The exocarp had more DAMs common to all developmental stages. The common DAMs identified at each developmental stage contained some metabolites enriched into the ‘Galactose metabolism’ pathway, such as D-fructose, D-tagatose, alpha-D-glucose, and D-mannose. Coincidentally, KEGG enrichment analysis of DAMs at each stage was mainly concentrated in the ‘Galactose metabolism’ pathway. Despite the lack of research on the role of galactose in exocarp texture, the intensity and distribution of immunofluorescence signals on apples showed that galactose and arabinose were the main reasons for the higher hardness of ‘Hanfu’ [24]. It is worth noting that the texture of most apples, whether described as soft or hard, refers to the flesh formed from the fusion of the hypanthium and the ovary [1]. In contrast, the edible part of jujube is the exocarp and mesocarp in the real fruit. The critical role of galactose metabolism in texture may be similar, although the sources of edible parts of fruits of the two species are different. In these DAMs enriched in the ‘Galactose metabolism’ pathway, D-tagatose and D-fructose were expressed higher in the exocarp of ‘P15’. In addition to these common DAMs, other DAMs screened at a particular stage also showed an accumulation pattern consistent with texture characteristics. D-galacturonic acid (GalA) showed higher expression in the exocarp of ‘P15’. This result is consistent with the grape mesocarp research that showed that hard phenotype grapes may provide more D-galactose-derived GalA, although the fruit states of these two species are different [25]. GalA could be derived from D-galactose, D-glucose, and D-glucuronic acid. GalA is mainly enriched in ‘Pentose and glucuronate interconversions’ and ‘Ascorbate and aldarate metabolism’ pathways. Ascorbate affects the cross-linking of cell wall components, and its degradation produces hydroxyl radicals that degrade pectin and cellulose [26]. Compared to the exocarp, there were only five common DAMs at the four stages of mesocarp development. However, D-mannose, which is related to galactose metabolism, was one of them. The ‘Galactose metabolism’ pathway was still the pathway of DAM enrichment at each stage.

These DEGs identified in the exocarp and mesocarp include some genes related to cell wall polysaccharides. However, it was surprising to find that many related genes were not present in the commonly expressed DEGs. This could be due to the fact that these genes are only expressed at specific developmental stages. Like the metabolomic, many DEGs were enriched in the ‘Galactose metabolism’ pathway. We mainly focused on genes related to cell wall polysaccharides and the ‘Galactose metabolism’ pathway after considering the K-means clustering analysis, KEGG pathway combined analysis and the O2PLS model. These related genes include cell wall component degrading enzymes *β-Gal*, *PL*, *β-Glu*, and cell wall component synthetase *CesA*. 

β-galactosidase (β-Gal) is a constituent enzyme related to cell wall galactose metabolism during fruit growth and development. It is a catalyst for regulating galactose degradation during senescence. It can degrade cell wall polysaccharides containing galactosides, such as pectin and cellulose, and release free galactose [27]. In two apple cultivars with differences in texture, *β-Gal* genes were expressed higher in softer cultivars [28]. In the strawberry, antisense expression of the *Faβ-Gal4* showed a 30% increase in fruit hardness [13]. The three *β-Gal* genes with a high correlation with DAMs in the ‘Galactose metabolism’ pathway, both in the exocarp and mesocarp, were expressed higher in ‘Dongzao’. Their high expression may be related to the lower hardness of ‘Dongzao’. The role of the *β-Gal* gene in strawberry fruit is to make it soft at ripening, while in apple and melon, it reduces the hardness, but the fruit is still hard and brittle. Jujube fruit remains hard and crisp at maturity, but it becomes soft at the post-ripening stage. It is worth exploring whether or not the role of the *β-Gal* gene in different ripening stages of jujube fruit is different.

Pectin lyase (PL) depolymerizes pectin via a β-elimination reaction mechanism and degrades pectin macromolecules via α-1,4-glycosidic bond breakage [29]. When the expression of tomato *PL* gene *SlPL* was inhibited by RNAi or CRISPR, the firmness of the fruit was significantly enhanced [30,31]. The latest research shows that using VIGS technology on the peach instantaneously silenced *PpePL1* and *PpePL15*, and the transgenic fruit maintained a greater hardness than the control group did in the early storage stage [14]. In our research, the *PL* gene screened out from the exocarp was expressed higher in ‘Dongzao’, which may contribute to the low firmness of ‘Dongzao’ fruit. It is worth noting that for fruits such as apples and strawberries, which develop from the hypanthium and receptacle, and for fruits such as peaches, which develop from the ovary, the effect of these genes on texture is primarily observed in the flesh or mesocarp. For jujube fruit, in which the exocarp and mesocarp are both edible parts, the effect of these genes on exocarp texture is also worthy of attention.

β-glucosidase (β-Glu) is an essential enzyme for cellulose degradation, which converts cellulosic disaccharides into glucose [32]. Cellulose synthase (CesA) catalyzes the assembly of β-1,4 glucan polymers into protofibrils, which are subsequently transformed into cellulose microfibrils [33]. In our research, four *β-Glu* genes were found to be expressed at higher levels in ‘Dongzao’, which could contribute to the degradation of cellulose and result in a softer texture compared to that of ‘P15’. The slightly higher expression of the *CesA* gene in ‘P15’ may affect the texture of the mesocarp by promoting cellulose synthesis. 

Transcription factors are a kind of protein with a unique structure that can activate or inhibit target genes. The regulation of transcription factors on metabolite accumulation is worthy of special attention. In Musa acuminata, *MaTCP20* can combine with *MaTCP5* and promote the transcription of *XTH10/11* (xyloglucan glycosyltransferase/hydrolase) to lead to the softening of the fruit [34]. The two *TCP* genes found in our research show an opposite expression pattern. evm.model.Contig69.109 was expressed higher in ‘Dongzao’, which is consistent with the results found for bananas. It may be similar to the regulation pattern of the *TCP* gene in banana. The same situation was found in *WRKY* and *MYB* genes. These genes exhibited different expression patterns, which may regulate other downstream genes. One *MYB* gene, evm.model.Contig11.191, was identified as a significant variable in the O2PLS analysis of the exocarp (Table S4). This gene was more highly expressed in ‘Dongzao’, and its downstream regulatory genes deserve further investigation. *SCAP1*, a member of the *DOF* gene family, regulates the expression of pectin methyl esterase [35]. The banana DOF transcription factor *MaDof23* transcriptionally inhibited the expression of *MaEXP1/2/3/5*, *MaXET7*, *MaPG1*, *MaPME3*, and *MaPL2* [36]. The *DOF* gene (evm.model.Contig69.54) was expressed higher in ‘Dongzao’, and its downstream genes and regulatory pattern deserve attention. In melon, the fruit firmness caused by the overexpression of the *CmbHLH32* gene was significantly lower than that of the wild type [37]. The evm.model.Contig13.193 gene was found in both the exocarp and mesocarp and had a strong relationship with several key DAMs. The expression of this *bHLH* gene was higher in the relatively soft cultivar ‘Dongzao’, which was similar to the results found for melon. This may indicate that jujube and melon have the same texture regulation mechanism. The specific role of these transcription factors in fruit texture formation warrants further study. 

**Figure 5:**
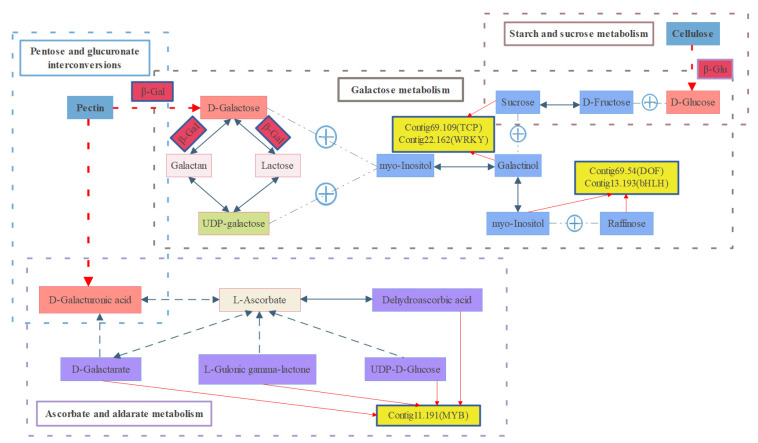
Metabolomic and transcriptomic profiling of the texture of flesh jujube fruit.

The ‘Galactose metabolism’ pathway is one of the critical pathways leading to the different texture traits of fresh jujube fruit. We constructed a network diagram linking key metabolites and genes (Figure 5). The galactose residues on the pectin skeleton are decomposed into free galactose by *β-Gal* and then converted to UDP-galactose with the help of *β-Gal* and other genes. Galactinol can be created from UDP-galactose, galactose, and myo-inositol. Cellulose is eventually degraded into glucose by *β-Glu*. Together with fructose, glucose can be converted into sucrose. This part also involves the ‘Starch and sucrose metabolism’ pathway. Sucrose with galactinol can turn into myo-inositol and raffinose. *TCP*, *WRKY*, *DOF*, and *bHLH* may be involved in regulating these metabolic processes. On the other hand, pectin is finally degraded into galacturonic acid under the action of various degrading enzymes. This step takes place in the ‘Pentose and glucuronate interconversions’ pathway. Galacturonic acid and ascorbate can be interconverted through multiple metabolites. *MYB* may regulate ascorbate-centered dehydroascorbic acid, D-galactarate, L-gulonic gamma-lactone, and UDP-D-glucose-related metabolic processes. By exploring the functions of these genes and transcription factors related to cell wall metabolism, it may be possible to better understand and regulate the texture of fruit. Further studies are necessary to fully uncover the role of these genetic factors in texture formation.

## 4. Materials and Methods

### 4.1. Plant Materials

The trees of *Z. jujuba* ‘Dongzao’ and ‘P15’ were grown at the National Key Base for Improved Chinese Jujube Cultivar (Cangzhou, China), under similar management conditions. Fruit samples were collected from four fruit development stages, i.e., the enlargement stage (40 DAF, E), green mature stage (80 DAF, G), white mature stage (100 DAF, W), and half-red stage (120 DAF, H) [38]. For each stage, three biological replicates of the samples were prepared, each of which comprised thirty healthy fruits. After texture analysis, the exocarp (E) and mesocarp (M) were separated, cut into small pieces and stored at −80 °C after being quickly frozen in liquid nitrogen.

### 4.2. Fruit Texture Detection

The firmness of the exocarp and mesocarp was determined using the TA.XT Plus Texture Analyzer (UK Stable Micro Systems) instrument. The P/2 probe was selected and the test parameters were modified based on previous studies [5]. Thirty fruits were used to set the plural number, ensuring that the result was representative.

### 4.3. Metabolite Profiling Using LC-MS/MS and Data Analysis

For metabolite determination, frozen samples were ground into a fine powder using liquid nitrogen. All processes were performed according to the non-targeted metabolomic analysis process of ultra-performance liquid chromatography-Q-TOF MS. The original data were transformed using ProteoWizard. The XCMS program was used for peak alignment, retention time correction, and peak area extraction. The metabolite structures were identified via accurate mass number matching (<25 PPM) and secondary spectrogram matching [39,40]. 

The principal component analysis (PCA) model was obtained by Pareto-scaling the peaks extracted from all the experimental and quality control (QC) samples. According to the variable importance for the project (VIP) obtained by the OPLS-DA model, the influence of the intensity and interpretation ability of the expression patterns of the metabolite on the classification and discrimination of each group of samples were measured. VIP > 1 and *p* value < 0.1 indicated differentially accumulated metabolites (DAMs). Both expression heat maps and differential Venn diagrams were generated using R software version 3.6.2. Kyoto Encyclopedia of Genes and Genomes (KEGG) was used to calculate the significance level of the metabolite enrichment of each pathway to identify significantly affected metabolic and signal transduction pathways.

### 4.4. Transcriptome Sequencing and Data Analysis

All samples were consigned to Tianjin Novogene Co., Ltd. for total RNA extraction and sequencing using Illumina Genome Analyzer. Clean data were obtained by removing adapters and low-quality sequences. After that, the Q30 and GC contents of clean data were calculated. The clean reads were mapped to the reference jujube genome ‘Junzao’ [41]. HTSeq V0.6.1 was used to calculate readings mapped to each gene, and DESeq2 was used to identify expression differences between sample groups. Based on the comparison of fragments per kilobase of transcript per million (FPKM) values, the genes with Padj < 0.01 and |log2Ratio| ≥ 2 were defined as differential expression genes (DEGs). Gene ontology (GO) and KEGG were also analyzed.

### 4.5. Combined Metabolomic and Transcriptomic Analyses

K-means clustering was used to analyze the different expression patterns of and co-regulatory relationships between metabolomics and transcriptomics. Bidirectional orthogonal partial least squares (O2PLS) analysis was used to evaluate their intrinsic correlation. Pearson correlation analysis between critical DAMs and DEGs was performed using the R software.

## 5. Conclusions

Based on metabolomics analysis and transcriptome sequencing, the exocarp and mesocarp have different metabolites and genes controlling texture formation. However, this mainly regards cell wall materials and related genes. The integration of multiple strategies for the analysis of both metabolomics and transcriptomics revealed that the ‘Galactose metabolism’ pathway was the key to the texture differences in fresh jujube fruit, and related metabolites can be used as indicators to quantify the texture of jujube fruit. *β-Gal*, *MYB*, *DOF*, *bHLH*, and other genes may play an essential role in regulating the texture of fresh jujube fruit. These results provide new insights into the factors affecting the texture of fresh jujube fruit.

## Figures and Tables

**Table 1:** Overlapping KEGG pathways in the joint metabolomic and transcriptomic analysis of the exocarp.

Stages	ID	Names
Enlargement stage	map00073	Cutin, suberine and wax biosynthesis
	map00410	beta-alanine metabolism
Green mature stage	map00052	Galactose metabolism
	map00630	Glyoxylate and dicarboxylate metabolism
	map02010	ABC transporters
	map00350	Tyrosine metabolism
White mature stage	map00053	Ascorbate and aldarate metabolism
	map00052	Galactose metabolism
	map00051	Fructose and mannose metabolism
	map00908	Zeatin biosynthesis
Half-red stage	map00052	Galactose metabolism
	map02010	ABC transporters
	map00073	Cutin, suberine and wax biosynthesis
	map00561	Glycerolipid metabolism

**Table 2:** DAMs and DEGs with high correlation in the exocarp.

DEG	Annotation	DAM	Pathway	Pearson’s r
evm.model.Contig21.3.80	*β-Gal*	D-Fructose	Fructose and mannose metabolism	−0.80
			Galactose metabolism	
			Starch and sucrose metabolism	
		Galactinol	Galactose metabolism	−0.81
evm.model.Contig67.34	*β-Gal*	Sucrose	Galactose metabolism	−0.80
			Starch and sucrose metabolism	
		Galactinol	Galactose metabolism	−0.84
evm.model.Contig67.23	*β-Gal*	Galactinol	Galactose metabolism	−0.84
evm.model.Contig87.33	*β-Glu*	Galactinol	Galactose metabolism	−0.81
		D-Fructose	Fructose and mannose metabolism	−0.82
			Galactose metabolism	
			Starch and sucrose metabolism	
evm.model.Contig87.32	*β-Glu*	Galactinol	Galactose metabolism	−0.81
		D-Fructose	Fructose and mannose metabolism	−0.81
			Galactose metabolism	
			Starch and sucrose metabolism	
evm.model.Contig36.473	*β-Glu*	Sucrose	Galactose metabolism	−0.81
			Starch and sucrose metabolism	
evm.model.Contig2.294	*PL*	Sucrose	Galactose metabolism	−0.85
			Starch and sucrose metabolism	
		Galactinol	Galactose metabolism	−0.85
evm.model.Contig10.1.49	*TCP*	D-galacturonic acid	Pentose and glucuronate interconversions	0.95
			Ascorbate and aldarate metabolism	
evm.model.Contig69.109	*TCP*	Sucrose	Galactose metabolism	−0.88
			Starch and sucrose metabolism	
		Galactinol	Galactose metabolism	−0.91
evm.model.Contig22.162	*WRKY*	Sucrose	Galactose metabolism	−0.87
			Starch and sucrose metabolism	
		Galactinol	Galactose metabolism	−0.92
evm.model.Contig22.167	*WRKY*	D-galacturonic acid	Pentose and glucuronate interconversions	0.99
			Ascorbate and aldarate metabolism	
evm.model.Contig11.191	*MYB*	L-Gulonic gamma-lactone	Ascorbate and aldarate metabolism	0.91
		D-Galactarate	Ascorbate and aldarate metabolism	0.94
		Dehydroascorbic acid	Ascorbate and aldarate metabolism	0.94
evm.model.Contig12.1.173	*MYB*	D-Mannose 1-phosphate	Fructose and mannose metabolism	0.81
		Raffinose	Galactose metabolism	0.82
evm.model.Contig57.486	*AP2/ERF*	Alpha-D-Glucose	Fructose and mannose metabolism	−0.86
			Galactose metabolism	
evm.model.Contig73.79	*AP2/B3*	D-galacturonic acid	Pentose and glucuronate interconversions	0.99
			Ascorbate and aldarate metabolism	
evm.model.Contig12.1.96	*GATA*	D-galacturonic acid	Pentose and glucuronate interconversions	0.85
			Ascorbate and aldarate metabolism	
		D-Galactarate	Ascorbate and aldarate metabolism	0.84
		Dehydroascorbic acid	Ascorbate and aldarate metabolism	0.81
		L-Gulonic gamma-lactone	Ascorbate and aldarate metabolism	0.83
evm.model.Contig69.54	*DOF*	Raffinose	Galactose metabolism	0.82
evm.model.Contig13.193	*bHLH*	Raffinose	Galactose metabolism	0.80

**Table 3:** Overlapping KEGG pathways in the joint metabolomic and transcriptomic analysis of the mesocarp.

Stages	ID	Names
Enlargement stage	map00410	beta-Alanine metabolism
	map01040	Biosynthesis of unsaturated fatty acids
Green mature stage	map00630	Glyoxylate and dicarboxylate metabolism
White mature stage	map02010	ABC transporters
	map00052	Galactose metabolism
Half-red stage	map00052	Galactose metabolism
	map00053	Ascorbate and aldarate metabolism
	map02010	ABC transporters
	map00330	Arginine and proline metabolism
	map00908	Zeatin biosynthesis
	map00020	Citrate cycle (TCA cycle)

**Table 4:** DAMs and DEGs with high correlation in the mesocarp.

DEG	Annotation	DAM	Pathway	Pearson’s r
evm.model.Contig109.23	*CesA*	α-D-Galactose 1-phosphate	Galactose metabolism	0.93
evm.model.Contig87.31	*β-Glu*	α-D-Galactose 1-phosphate	Galactose metabolism	−0.82
evm.model.Contig84.454	*ERF*	L-(-)Sorbose	Fructose and mannose metabolism	0.82
		Raffinose	Galactose metabolism	0.93
		Isomaltose	Starch and sucrose metabolism	0.81
evm.model.Contig42.394	*MADS-box*	UDP-D-Glucose	Pentose and glucuronate interconversions	−0.85
			Galactose metabolism	
			Ascorbate and aldarate metabolism	
			Starch and sucrose metabolism	
		L-(-)Sorbose	Fructose and mannose metabolism	−0.92
		Galactinol	Galactose metabolism	−0.93
		Sucrose	Galactose metabolism	−0.92
			Starch and sucrose metabolism	
		Isomaltose	Starch and sucrose metabolism	−0.93
evm.model.Contig51.23	*AP2/B3*	UDP-D-Glucose	Pentose and glucuronate interconversions	−0.87
			Galactose metabolism	
			Ascorbate and aldarate metabolism	
			Starch and sucrose metabolism	
		Galactinol	Galactose metabolism	−0.83
		Sucrose	Galactose metabolism	−0.81
			Starch and sucrose metabolism	
		L-Gulonic gamma-lactone	Ascorbate and aldarate metabolism	0.88
evm.model.Contig11.191	*MYB*	UDP-D-Glucose	Pentose and glucuronate interconversions	−0.93
			Galactose metabolism	
			Ascorbate and aldarate metabolism	
			Starch and sucrose metabolism	
		L-Gulonic gamma-lactone	Ascorbate and aldarate metabolism	0.82
evm.model.Contig69.54	*DOF*	D-Mannose	Fructose and mannose metabolism	0.94
			Galactose metabolism	
		myo-Inositol	Galactose metabolism	0.94
			Ascorbate and aldarate metabolism	
		D-Tagatose	Galactose metabolism	0.91
evm.model.Contig13.193	*bHLH*	D-Mannose	Fructose and mannose metabolism	0.90
			Galactose metabolism	
		myo-Inositol	Galactose metabolism	0.88
			Ascorbate and aldarate metabolism	
		D-Tagatose	Galactose metabolism	0.85

## Data Availability

The datasets used or analyzed during the study are available from the corresponding author upon reasonable request. The raw reads of the transcriptome datasets were deposited in the NCBI Sequence Read Archive database (PRJNA954530).

## Supplementary Materials

The following supporting information can be downloaded at https://www.mdpi.com/article/10.3390/plants12112087/s1. Figure S1: Total ion chromatogram overlapping pattern of QC sample. QC 1–5 indicates *n* = 5 test per modes; (a) positive-ion modes, and (b) negative-ion modes. QC: quality control. Figure S2: Distribution and classification of all DAMs. Figure S3: The situation of DAMs screened out from exocarp; (a) upregulation and downregulation of DAMs at different developmental stages. (b) Venn diagram comparing metabolites at different developmental stages. D: ‘Dongzao’. P: ‘P15’. E: exocarp. M: mesocarp. E: enlargement. G: green mature stage. W: white mature stage. H: half-red stage. Figure S4: Top 20 enriched KEGG pathways of DAMs in exocarp. (a) Enlargement stage. (b) Green mature stage. (c) White mature stage. (d) Half-red stage. Figure S5: The situation of DAMs screened out from the mesocarp. (a) Upregulation and downregulation of DAMs at different developmental stages. (b) Venn diagram comparing metabolites at different developmental stages. D: ‘Dongzao’. P: ‘P15’. E: exocarp. M: mesocarp. E: enlargement. G: green mature stage. W: white mature stage. H: half-red stage. Figure S6: Top 20 enriched KEGG pathways of DAMs in exocarp. (a) Enlargement stage. (b) Green mature stage. (c) White mature stage. (d) Half-red stage. Figure S7: The situation of DEGs screened out from the exocarp. (a) Upregulation and downregulation of DEGs at different developmental stages. (b) Venn diagram comparing genes at different developmental stages. D: ‘Dongzao’. P: ‘P15’. E: exocarp. M: mesocarp. E: enlargement. G: green mature stage. W: white mature stage. H: half-red stage. Figure S8: Analysis of GO terms enriched for the DEGs in the exocarp. (a) Enlargement stage. (b) Green mature stage. (c) White mature stage. (d) Half-red stage. Figure S9: Top 20 enriched KEGG pathways of DEGs in the exocarp. (a) Enlargement stage. (b) Green mature stage. (c) White mature stage. (d) Half-red stage. Figure S10: The situation of DEGs screened out from the mesocarp. (a) Upregulation and downregulation of DEGs at different developmental stages. (b) Venn diagram comparing genes at different developmental stages. D: ‘Dongzao’. P: ‘P15’. E: exocarp. M: mesocarp. E: enlargement. G: green mature stage. W: white mature stage. H: half-red stage. Figure S11: Analysis of GO terms enriched for the DEGs in the mesocarp. (a) Enlargement stage. (b) Green mature stage. (c) White mature stage. (d) Half-red stage; Figure S12: Top 20 enriched KEGG pathways of DEGs in the mesocarp. (a) Enlargement stage. (b) Green mature stage. (c) White mature stage. (d) Half-red stage; Figure S13: K-means analysis of DAMs and DEGs in the exocarp. D: ‘Dongzao’. P: ‘P15’. E: enlargement. G: green mature stage. W: white mature stage. H: half-red stage. Figure S14: Load diagram of the first 25 variables In the exocarp. Figure S15: K-means analysis of DAMs and DEGs in mesocarp. D: ‘Dongzao’. P: ‘P15’. E: enlargement. G: green mature stage. W: white mature stage. H: half red stage. Figure S16: Load diagram of the first 25 variables in the mesocarp. D: ‘Dongzao’. P: ‘P15’. E: enlargement. G: green mature stage. W: white mature stage. H: half-red stage. Table S1: List of metabolites identified in this study; Table S2A: The information about all DAMs in the exocarp; Table S2B: The information about all DAMs in the mesocarp; Table S3: An overview of the RNA-Seq data; Table S4: Top 25 loading elements of O2PLS analysis in the exocarp; Table S5: Top 25 loading elements of O2PLS analysis in the mesocarp.
