# Supplementary material for: The Key Metabolic Network and Genes Regulating the Fresh Fruit Texture of Jujube (Ziziphus jujuba Mill.) Revealed via Metabolomic and Transcriptomic Analysis
Source: Plants (Basel). 2023 May 24;12(11):2087. doi: 10.3390/plants12112087 (PMC10255913; doi:10.3390/plants12112087)
Supplement: Supplementary file 1 [file plants-12-02087-s001.zip › Supplementary Materials-Figures.pdf]

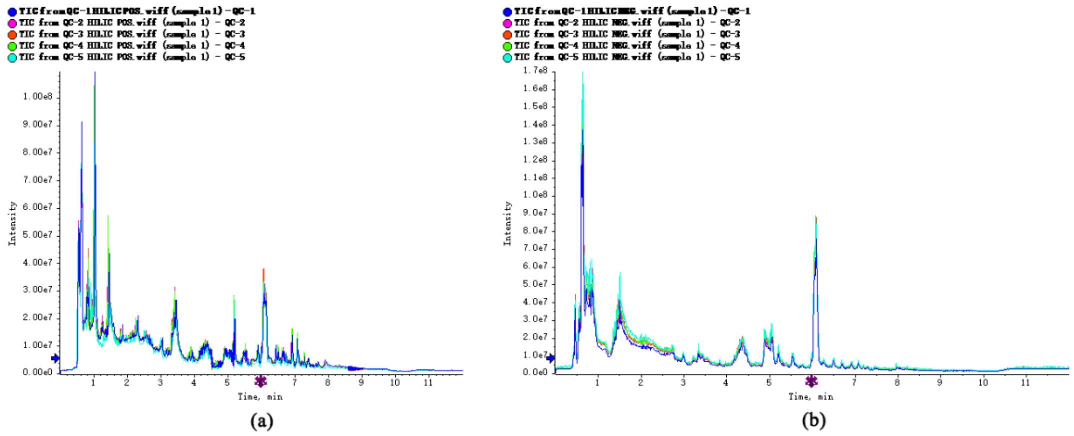

Figure S1: Total ion chromatogram overlapping pattern of QC sample. QC 1-5 indicates n=5 test per modes. (a) positive ion modes. (b) negative ion modes. QC: quality control.

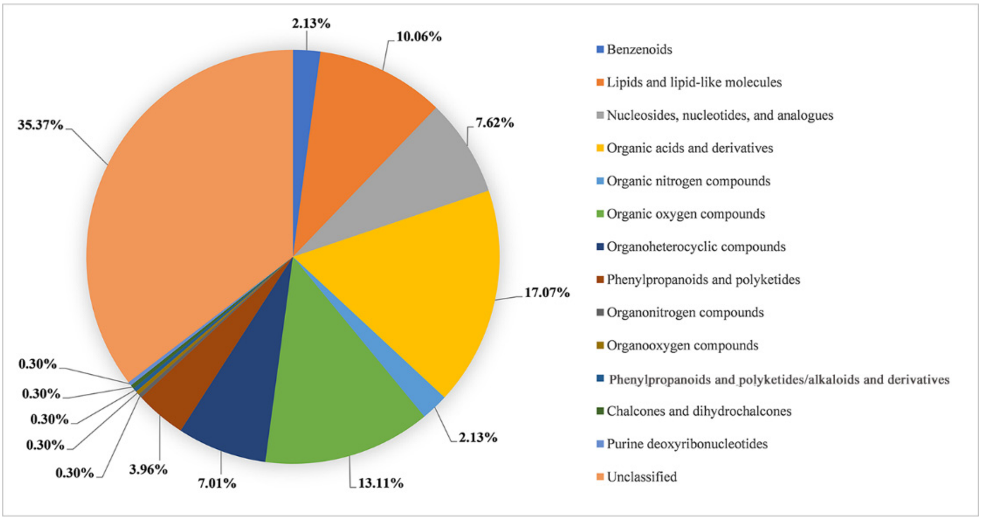

Figure S2: Distribution and classification of all DAMs.

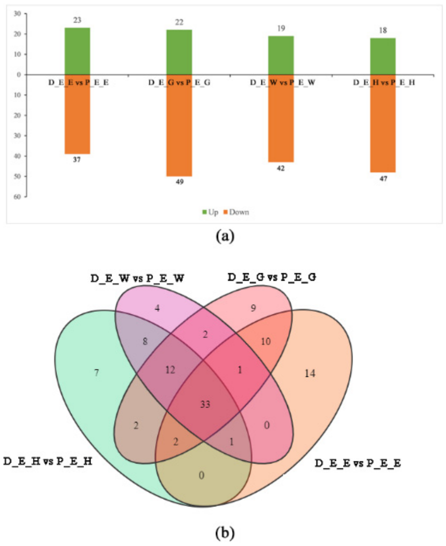

Figure S3: The situation of DAMs screened out from exocarp. (a) Up-regulation and down-regulation of DAMs at different developmental stages. (b) Venn diagram comparing me-tabolites at different developmental stages. D: ‘Dongzao’. P: ‘P15’. E: exocarp. M: mesocarp. E: enlargement. G: green mature stage. W: white mature stage. H: half red stage.

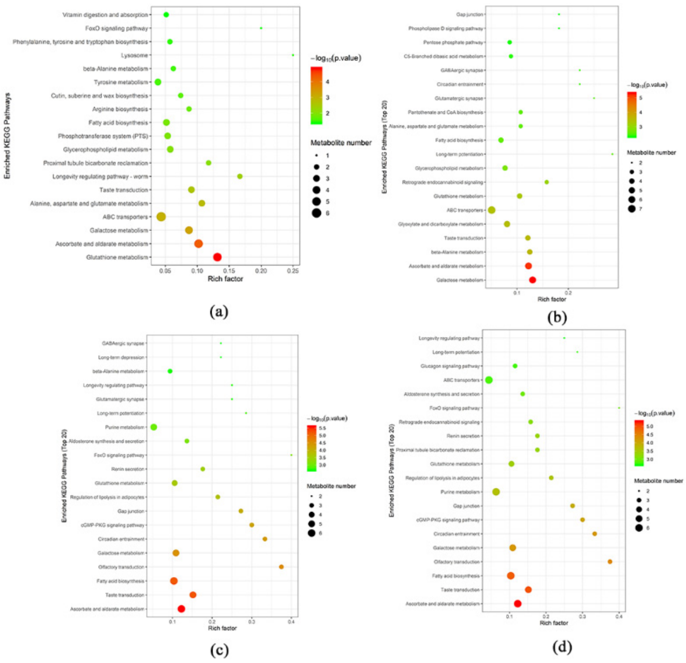

Figure S4: Top 20 enriched KEGG pathways of DAMs in exocarp. (a) Enlargement stage. (b) Green mature stage. (c) White mature stage. (d) Half red stage.

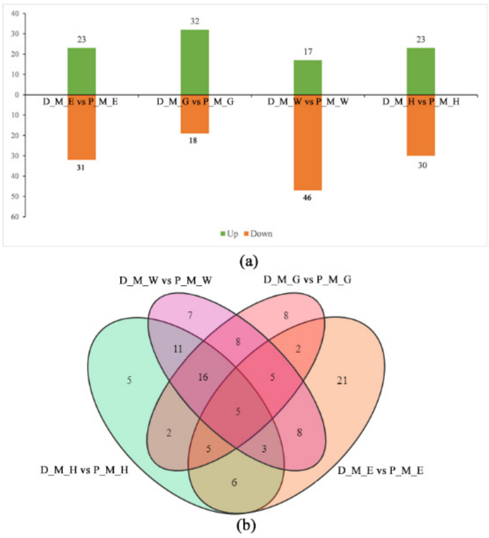

Figure S5: The situation of DAMs screened out from mesocarp. (a) Up-regulation and down-regulation of DAMs at different developmental stages. (b) Venn diagram comparing me-tabolites at different developmental stages. D: 'Dongzao'. P: 'P15'. E: exocarp. M: mesocarp. E: enlargement. G: green mature stage. W: white mature stage. H: half red stage.

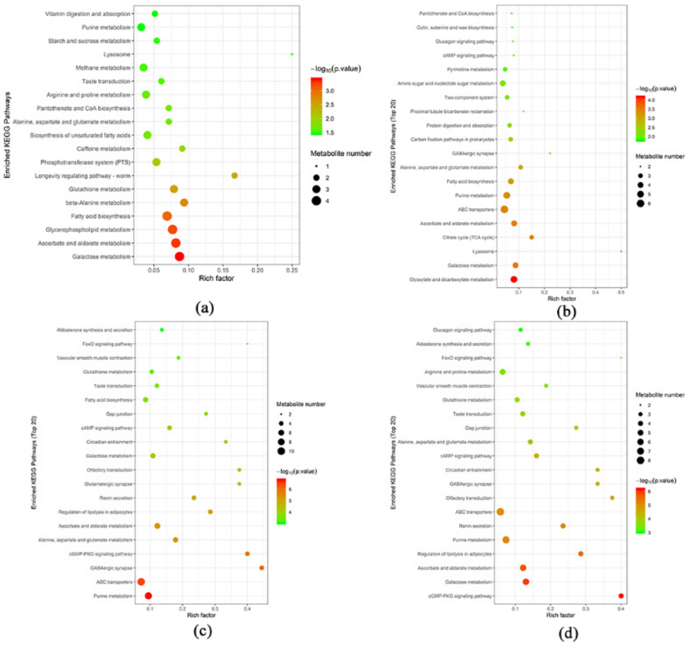

Figure S6: Top 20 enriched KEGG pathways of DAMs in exocarp. (a) Enlargement stage. (b) Green mature stage. (c) White mature stage. (d) Half red stage.

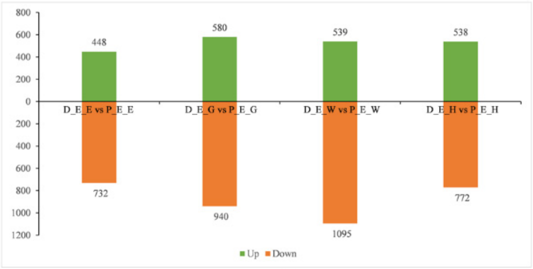

(a)

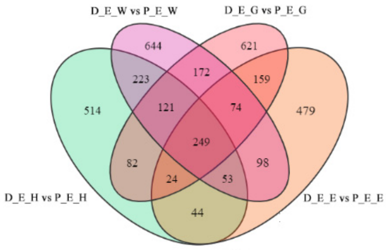

(b)

Figure S7: The situation of DEGs screened out from exocarp. (a) Up-regulation and down-regulation of DEGs at different developmental stages. (b) Venn diagram comparing genes at different developmental stage. D: ‘Dongzao’. P: ‘P15’. E: exocarp. M: mesocarp. E: enlargement. G: green mature stage. W: white mature stage. H: half red stage.

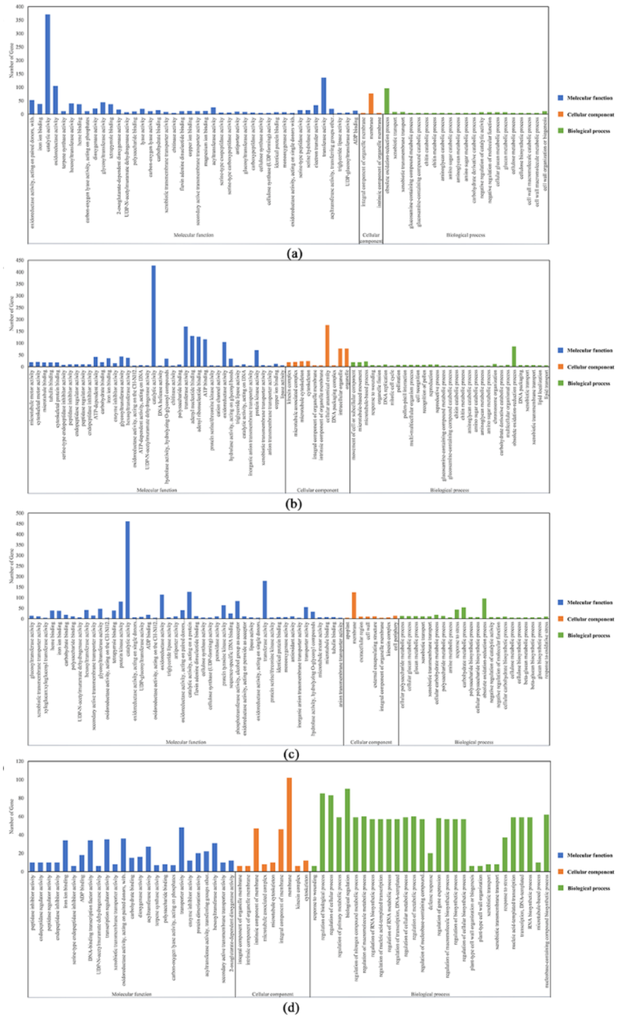

Figure S8: Analysis of GO terms enriched for the DEGs in the exocarp. (a) Enlargement stage. (b) Green mature stage. (c) White mature stage. (d) Half red stage.

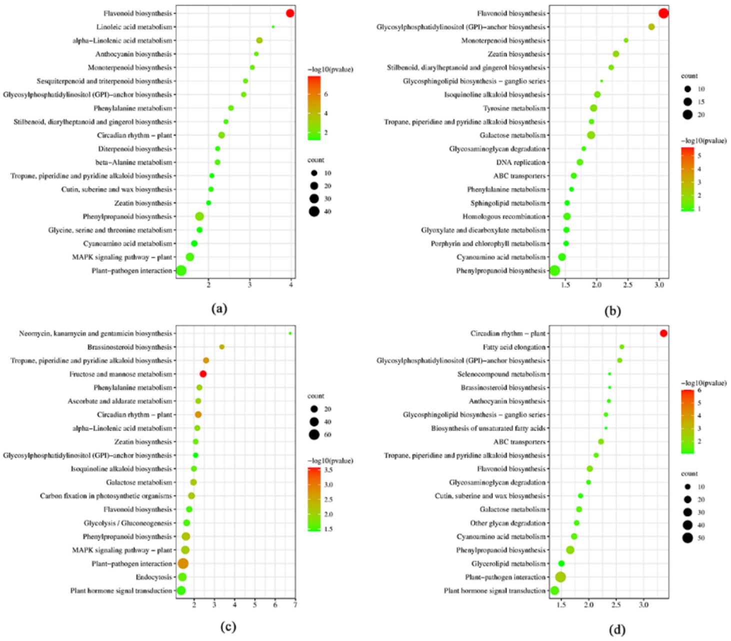

Figure S9: Top 20 enriched KEGG pathways of DEGs in exocarp. (a) Enlargement stage. (b) Green mature stage. (c) White mature stage. (d) Half red stage.

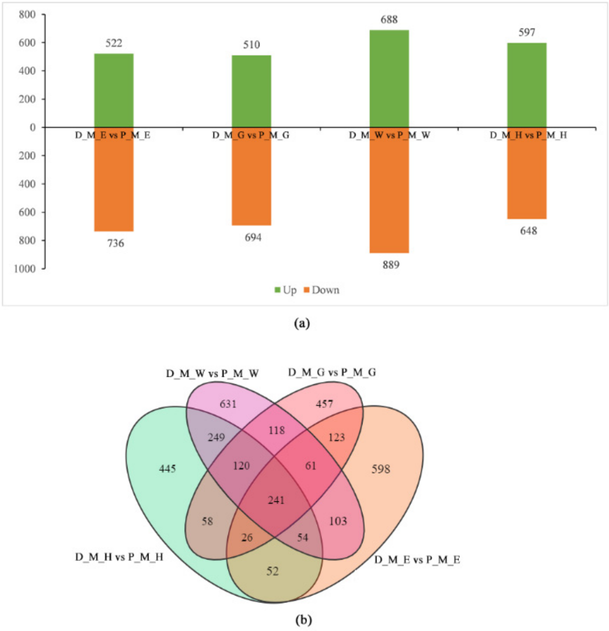

Figure S10: The situation of DEGs screened out from mesocarp. (a) Up-regulation and down-regulation of DEGs at different developmental stages. (b) Venn diagram comparing genes at different developmental stage. D: 'Dongzao'. P: 'P15'. E: exocarp. M: mesocarp. E: enlargement. G: green mature stage. W: white mature stage. H: half red stage.

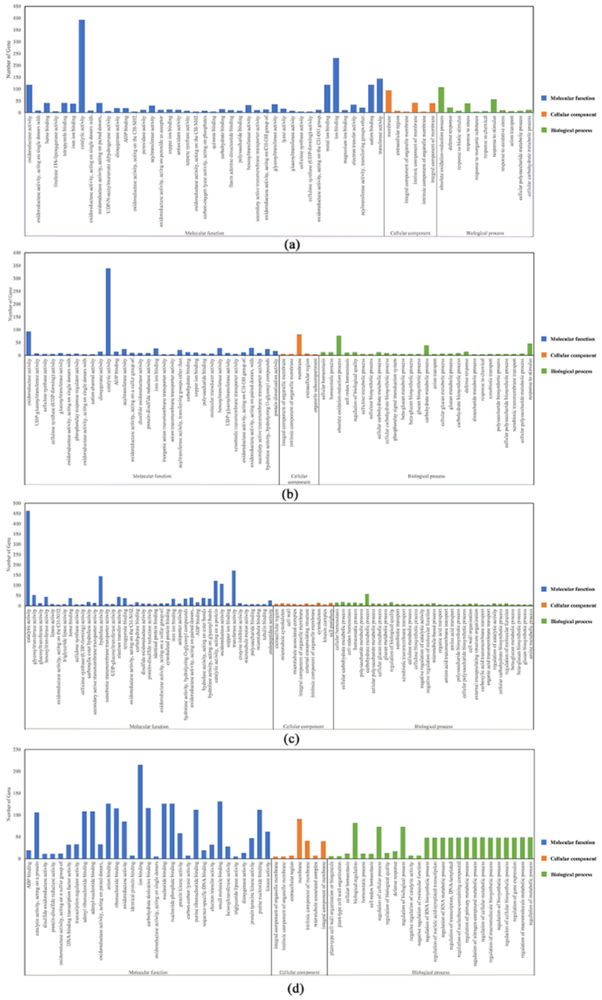

Figure S11: Analysis of GO terms enriched for the DEGs in the mesocarp. (a) Enlargement stage. (b) Green mature stage. (c) White mature stage. (d) Half red stage.

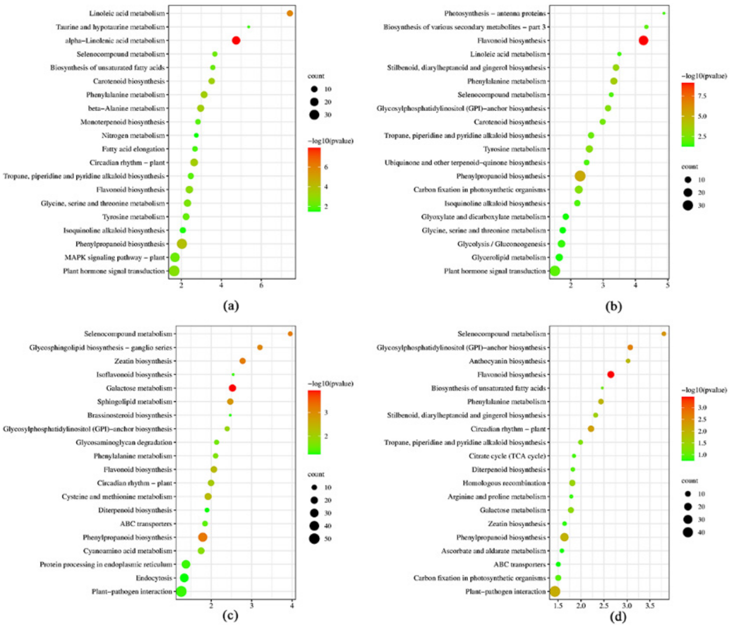

Figure S12: Top 20 enriched KEGG pathways of DEGs in mesocarp. (a) Enlargement stage. (b) Green mature stage. (c) White mature stage. (d) Half red stage.

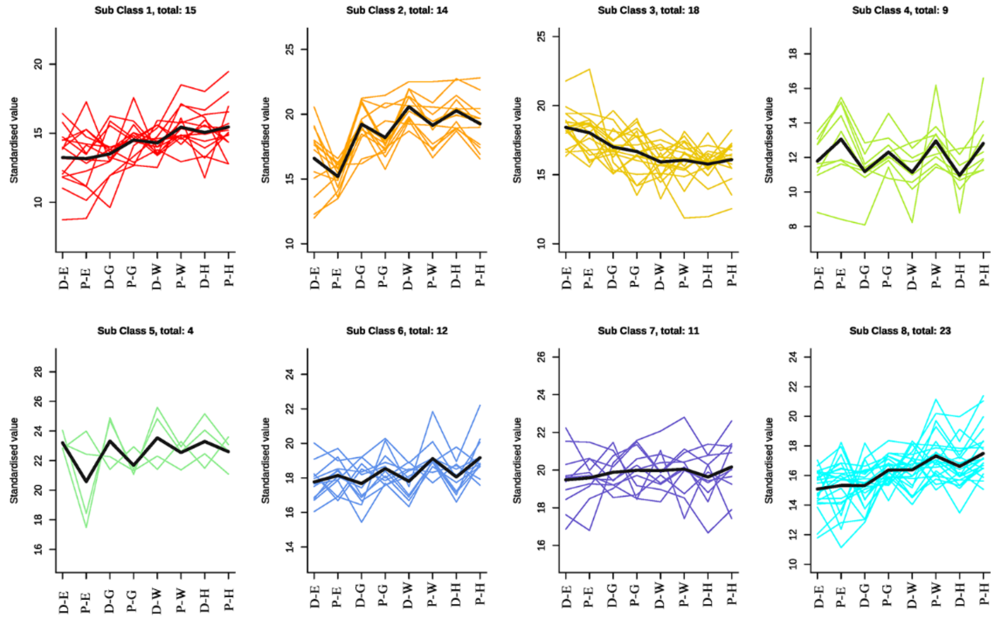

Figure S13: K-means analysis of DAMs and DEGs in exocarp. D: ‘Dongzao’. P: ‘P15’. E: enlargement. G: green mature stage. W: white mature stage. H: half red stage.

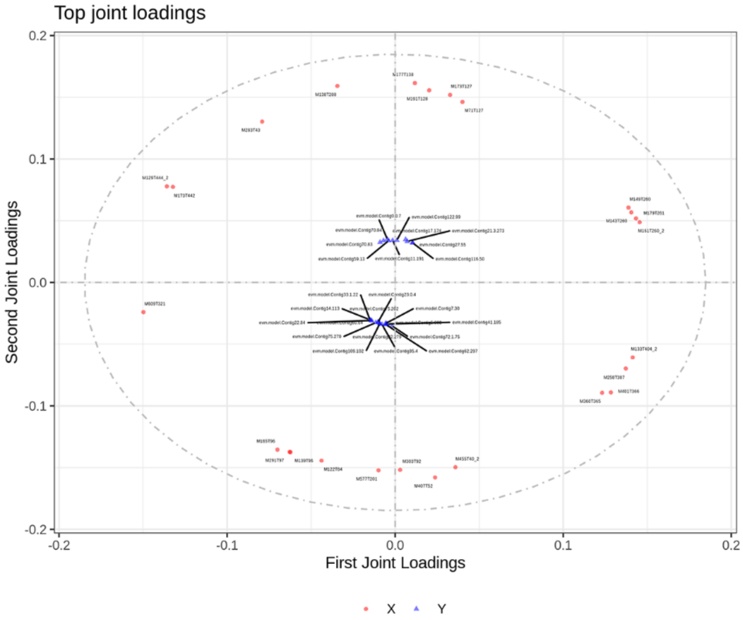

Figure S14: Load diagram of the first 25 variables in exocarp.

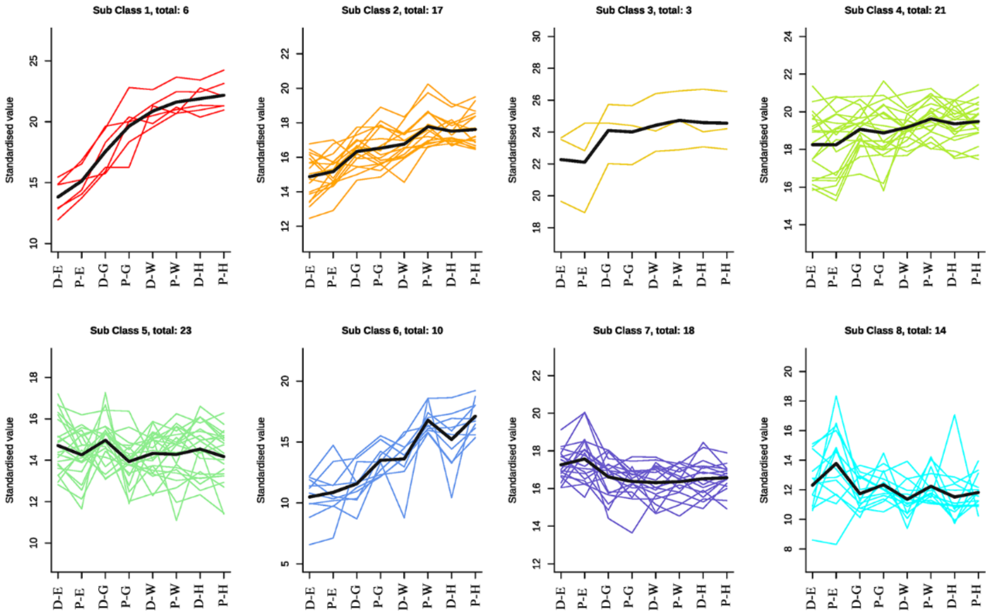

Figure S15: K-means analysis of DAMs and DEGs in mesocarp. D: 'Dongzao'. P: 'P15'. E: enlargement. G: green mature stage. W: white mature stage. H: half red stage.

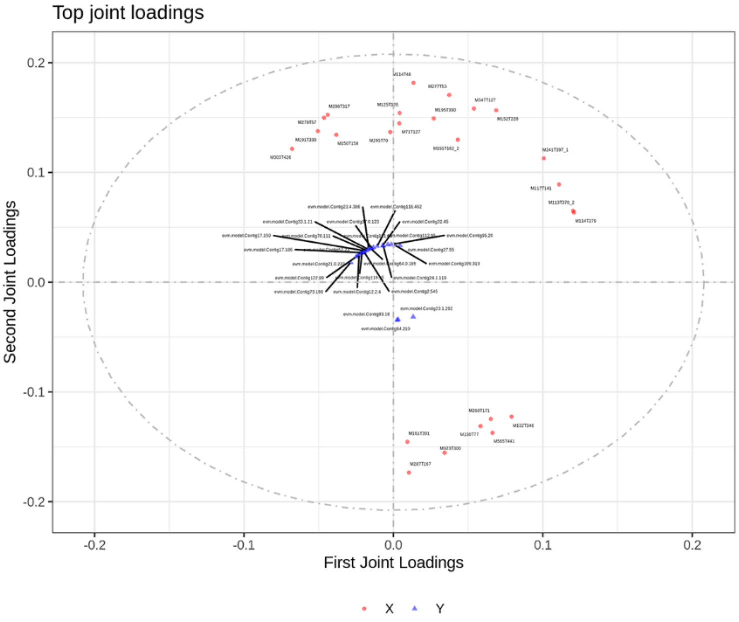

Figure S16: Load diagram of the first 25 variables in mesocarp. D: ‘Dongzao’. P: ‘P15’. E: en-largement. G: green mature stage. W: white mature stage. H: half red stage.
